# Positive affect dysregulation and its relation to binge eating size and frequency

**DOI:** 10.3389/fpsyg.2023.1146549

**Published:** 2023-05-22

**Authors:** Rebecca L. Flynn, Thomas A. Massion, Jacqueline A. Kosmas, Shannon R. Smith, Carli N. Mastronardi, Andrea K. Graham

**Affiliations:** ^1^Center for Behavioral Intervention Technologies, Northwestern University Feinberg School of Medicine, Chicago, IL, United States; ^2^Department of Medical Social Sciences, Northwestern University Feinberg School of Medicine, Chicago, IL, United States; ^3^Department of Psychiatry and Behavioral Sciences, Northwestern University Feinberg School of Medicine, Chicago, IL, United States; ^4^Department of Preventive Medicine, Northwestern University Feinberg School of Medicine, Chicago, IL, United States

**Keywords:** positive affect, negative affect, binge eating, subjective binge eating, objective binge eating, socio-demographic variables

## Abstract

Negative affect is an established predictor of binge eating, yet less is known about positive affect. Low positive affect has been theorized to increase binge eating, but a better understanding is needed on the relationship between positive affect and binge eating frequency and size. Participants were 182 treatment-seeking adults (76% self-identified as female; 45% self-identified their race as Black and 40% as White; and 25% self-identified their ethnicity as Hispanic/Latino) with self-reported recurrent binge eating (≥12 binge episodes in the past 3  months). Participants completed the positive and negative affect schedule (PANAS) survey and the eating disorder examination to assess frequency of objective binge episodes (OBEs) and subjective binge episodes (SBEs) over the past 3  months. OBEs and SBEs also were combined to yield total binge episodes over the past 3  months. Independent *t*-tests and linear regression analyses were used to test associations between positive affect scores and binge episode size and frequencies, and to compare low versus higher positive affect on binge frequency. Additional exploratory models were conducted controlling for negative affect, identity characteristics, and socio-demographic variables. Lower positive affect was significantly associated with more frequent total binge episodes, but not OBEs and SBEs when assessed independently. Findings remained consistent when controlling for covariates and when comparing individuals with the lowest versus higher positive affect levels. Overall, results lend support to the theory that low positive affect is associated with binge eating. Increasing positive affect may be an important treatment consideration for those with recurrent binge eating.

## Introduction

Binge eating is known to be influenced by varying levels of affect (Sultson et al., 2022). Negative affect is an established driver of binge eating (Dingemans et al., 2017), but less is known about positive affect. Mason et al. (2021a) have theorized that low positive affect increases risk of binge eating and binge eating disorder (BED) by directly and indirectly maintaining binge eating. More specifically, they propose that low positive affect directly predicts binge episodes, as well as indirectly through decreasing healthy behaviors such as physical activity, healthy eating, and social interaction. These latter factors are known to reduce the risk of binge eating (Neumark-Sztainer et al., 2006; Mason and Lewis, 2017), but may have the opposite effect when in deficit. Their model also is informed by research that has shown that higher baseline positive affect improves treatment outcomes among people with BED (Mason et al., 2021b,c).

Validating this theory requires additional research. For example, one area that would be useful to better understand is the relationship between positive affect and binge eating characteristics, such as binge episode frequency and binge size (e.g., small and large binge episodes). To date, most studies on the relationship between positive affect and binge eating have only comprised of individuals presenting with general binge episodes, regardless of size, or objective binge episodes (OBEs, i.e., larger binge episodes) and have used ecological momentary assessment (EMA). A 1 week EMA study, conducted with a sample of 111 men and women with BED, showed that low positive affect decreased prior to binge eating, then stabilized after (Schaefer et al., 2020). Results from this study reflect general binge episodes, as information on binge episode size was not collected (Schaefer et al., 2020). In another 1 week EMA study among 22 women with BED, Munsch et al. (2012) found that on binge eating days, positive affect significantly decreased prior to an objective binge episode and increased in the hours following an objective binge episode. Additionally, Smith et al. (2019) conducted a 2  week EMA study among 30 women who engaged in regular binge eating (≥1 OBE in the past month) and found that higher positive affect was protective against having OBEs in individuals with tendencies towards higher impulsive actions and lower impulsive choices. These studies provide support for the theory that low positive affect temporally drives general binge episodes, as well as episodes of OBEs; however, they do not capture the relationship with binge frequency, which is an important consideration as frequency is a measure of clinical severity (American Psychiatric Association, 2013).

Further, only one study, to our knowledge, has evaluated positive affect among individuals presenting with other types of binge eating, namely subjective binge episodes (SBEs, i.e., smaller binge episodes) and loss of control eating regardless of episode size (Van Malderen et al., 2019). This study examined how self-regulation and affectivity interact to contribute to binge eating in 301 adolescents who endorsed ≥1 OBE or SBE in the past month; results showed that positive affect was not associated with loss of control eating or OBEs, but the interaction between self-regulation and positive affect predicted the probability of SBEs (Van Malderen et al., 2019). More studies that investigate SBEs are needed because SBEs are clinically concerning and contribute to adverse symptoms like negative affect, body image concerns, and personality difficulties (Brownstone et al., 2013). Additionally, because the research on SBEs and positive affect is limited to adolescents, it will be useful to study this relationship in adults given that positive affect regulation changes with age (Stretton et al., 2022). Finally, extensions of the prior research would benefit from studying different binge episodes among people with recurrent binge eating over a longer duration (e.g., the past three months consistent with the clinical criterion; American Psychiatric Association, 2013) and based on clinical assessment versus self-report, which has been inconsistent in prior work.

Thus, the focus of this study is to explore the relation between positive affect and binge eating episode size and frequency among a sample of adults seeking intervention for binge eating and weight management. We also explored the impact of various socio-demographic characteristics, given that constructs like race, gender, and socioeconomic status interact with binge eating behaviors and binge eating risk factors (Napolitano and Himes, 2011; Reslan and Saules, 2011; West et al., 2019). Consistent with Mason and colleagues’ (2021a) model and the prior studies in this area, we hypothesized that lower positive affect would be associated with more frequent binge eating and associated with both SBEs and OBEs.

## Methods

This study is an exploratory analysis of baseline data collected as part of a clinical trial that aims to test the feasibility and preliminary efficacy of a digital intervention (“FoodSteps”) to help adults with comorbid recurrent binge eating (≥12 OBEs) and obesity [body mass index (BMI) ≥30 kg/m^2^] reduce binge eating and manage their weight. Data for these analyses included all individuals who completed a baseline assessment, including those who did not qualify for the subsequent trial [e.g., because their verified weight did not equate to a body mass index (BMI) ≥30 kg/m^2^, insufficient OBEs based on the clinical interview].

### Participants & procedures

Participants were recruited using flyers (e.g., in clinics), word of mouth referrals, online paid social media ads and free social media posts (i.e., on Craigslist, Facebook, and Instagram), and online research recruitment platforms (i.e., ResearchMatch, clinicaltrials.gov, The New Normal). Interested individuals (*N* = 1,557) completed online consent and an online screening questionnaire to determine initial eligibility. Potentially eligible participants were subsequently invited to complete a baseline assessment if, on the screener, they self-identified as non-pregnant, English-speaking, and an adult (≥18 years old) with obesity (BMI ≥30 kg/m^2^) and recurrent binge eating with distress (≥12 binge episodes during the past three months). BMI at screening was calculated based on self-reported height and weight. Binge eating at screening was assessed using items from the eating disorder diagnostic scale version 5 (Stice et al., 2000). To be invited to baseline, screen respondents also needed to be interested in reducing their binge eating and losing weight, have a valid email address, have access to a scale to measure their weight, and own a smartphone with a Wi-Fi connection and sufficient data plan to engage with the intervention. Individuals were excluded at screening if they were currently receiving services for binge eating or weight management, such as psychotherapy or a medically supervised weight loss program, or currently taking medication to manage their weight or binge eating for less than one month.

One hundred ninety-nine individuals completed the baseline assessment. The assessment was conducted remotely via Zoom and included verbal informed consent followed by an interview with a trained assessor; after the interview, participants were asked to complete an online survey comprised of several questionnaires. Participants were compensated $10 for completing the baseline assessment.

This study was approved by the Northwestern University Institutional Review Board.

### Measures

During the baseline assessment, participants were administered the diagnostic portion of the eating disorder examination (EDE) version 17, namely the sections on bulimic episodes, binge eating disorder, and compensatory behaviors (Cooper and Fairburn, 1987). For the purpose of this analysis, binge eating episodes are from these EDE data, not from self-report data at screening, and we focused on total frequency of OBEs and SBEs, as well as total binge episodes regardless of episode size, over the past three months.

The online survey included questions assessing participants’ demographics [including asking participants to take and upload a photograph of their weight while standing on a scale, consistent with procedures in other remotely-conducted trials (Leahey and Rosen, 2014) to verify weight and calculate BMI] and the positive and negative affect schedule (PANAS) (Watson et al., 1988). The PANAS is a 20-item questionnaire that asks respondents to indicate the extent to which they felt different mood states over the past 7  days. Each mood state is rated on a five-point Likert-like scale, ranging from 1 (very slightly or not at all) to 5 (extremely). The PANAS yields a positive affect subscale and a negative affect subscale, calculated by adding scores for the positive and negative affect items, respectively. Subscale scores range from 10–50, with higher scores representing higher affect ratings. The PANAS has demonstrated excellent factorial validity and adequate reliability across both subscales (Watson et al., 1988; Crawford and Henry, 2004). Cronbach alpha in our sample was 0.92 and 0.89 for the positive affect and negative affect items, respectively.

### Analyses

Of the 199 participants who completed the baseline interview, only 182 completed the PANAS, resulting in an analytic sample of 182.

To evaluate differences in positive affect by EDE binge episode size, participants were grouped based on the episode types they endorsed: OBEs only (*n* = 79, 43%), SBEs only (*n* = 32, 18%), or both OBEs and SBEs (*n* = 71, 39%). A one-way analysis of variance was conducted to explore differences in positive affect between these three groups.

We then evaluated the relationship between positive affect and binge frequency based on frequency of OBEs, frequency of SBEs, and frequency of total binge episodes regardless of episode size. Because of outliers, OBEs, SBEs, and total binge episodes were Winsorized to the 90^th^ percentile. For each of these three outcome variables, we first conducted linear regression analyses to explore the relationship between positive affect and binge frequency. Next, we ran a set of secondary exploratory linear regression models that evaluated positive affect on frequency of OBEs, SBEs, and total binge episodes controlling for covariates of negative affect (using the PANAS negative affect subscale), race, ethnicity, gender, income, and unemployment status, given their theoretical/clinical associations with binge eating. We assessed for collinearity by examining correlations between positive affect and subsequent covariates, in addition to verifying variance inflation factor (VIF) scores. Because there were very small numbers of participants in certain subgroups of these covariates, race only included those who identified as Black or White, and gender only included those who identified as male or female. Third, we conducted independent *t*-tests to explore differences in positive affect scores among those who endorsed binge eating above the clinical threshold (≥12 episodes in the past 3 months) compared to below the threshold (<12 episodes in the past 3 months). Finally, we conducted t-tests to explore whether positive affect dysregulation, characterized by endorsing low positive affect (≤lowest quartile on the PANAS positive affect subscale), was associated with more frequent binge eating compared to endorsing higher positive affect (>lowest quartile on the subscale). We operationalized positive affect dysregulation as *low* positive affect based on prior EMA study findings that indicate positive affect decreases prior to binge eating (Munsch et al., 2012; Schaefer et al., 2020). We also note that our cut-point, based on the lowest quartile, is consistent with scores ≤2 standard deviations below the mean reported in the original PANAS validation study (Watson et al., 1988), supporting our approach for how we operationalized low positive affect.

Analyses were conducted using SPSS software version 28. As analyses are exploratory, *p*-values <0.05 were considered statistically significant.

## Results

Sample characteristics of the 182 participants are presented in Table 1.

**Table 1:** Participant sample characteristics.

Characteristic	Full sample (*N* = 182)	OBEs only (*n* = 79)	SBEs only (*n* = 32)	OBEs + SBEs (*n* = 71)
Age, mean (SD)	42.51 (14.40)	39.42 (13.49)	48.63 (14.12)	43.18 (14.72)
BMI, mean (SD)	38.34 (7.88)	38.18 (8.65)	38.34 (5.48)	38.52 (7.80)
Income in US dollars, mean (SD)	75435.70 (80159.84)	67106.32 (50242.25)	94900.77 (141284.87)	77057.49 (74773.61)
Positive affect, mean (SD)	25.29 (8.93)	25.15 (9.37)	24.66 (8.65)	25.73 (8.66)
Negative affect, mean (SD)	30.70 (8.87)	32.37 (9.81)	30.52 (7.99)	28.90 (7.80)
Objective binge episodes, mean (SD)	31.21 (30.10)	47.62 (30.15)	—	27.03 (23.72)
Subjective binge episodes, mean (SD)	16.64 (20.55)	—	40.03 (19.08)	24.62 (17.41)
Total binge episodes, mean (SD)	51.07 (32.91)	—	—	51.68 (31.05)
*Gender, n (%)*				
Male	41 (23%)	23 (29%)	4 (13%)	14 (20%)
Female	138 (76%)	54 (69%)	28 (88%)	56 (79%)
Another gender	2 (1%)	1 (1%)	0 (0%)	1 (1%)
Prefer not to answer	1 (1%)	1 (1%)	0 (0%)	0 (0%)
*Race, n (%)*				
American Indian or Alaska Native	3 (2%)	0 (0%)	1 (3%)	2 (3%)
Asian	8 (4%)	6 (8%)	1 (3%)	1 (1%)
Black or African American	81 (45%)	37 (47%)	12 (38%)	32 (46%)
Native Hawaiian or other Pacific Islander	1 (1%)	0 (0%)	0 (0%)	1 (1%)
White	72 (40%)	29 (37%)	14 (44%)	29 (42%)
More than one race	14 (8%)	6 (8%)	4 (13%)	4 (6%)
Prefer not to answer	3 (2%)	1 (1%)	0 (0%)	2 (3%)
*Hispanic/Latino, n (%)*	45 (25%)	20 (26%)	9 (29%)	16 (23%)
*Employment status, n (%)*				
Employed	120 (66%)	50 (64%)	20 (65%)	50 (70%)
Unemployed	24 (13%)	12 (15%)	2 (6%)	10 (14%)
Disability	16 (9%)	10 (13%)	3 (10%)	3 (4%)
Retired	13 (7%)	2 (3%)	5 (16%)	6 (8%)
Prefer not to answer	9 (5%)	5 (6%)	2 (6%)	2 (3%)

BMI, body mass index, calculated based on weight from a photograph at baseline.

### Binge size

Mean (SD) positive affect scores by binge size groups were: SBEs only = 24.7 (8.7), OBEs only = 25.2 (9.4), and both = 25.7 (8.7). Mean positive affect scores did not significantly differ by binge size (*F* (2, 179) = 0.18; *p* = 0.84).

### Binge frequency

Positive affect was significantly associated with frequency of total binge episodes (*ß* = −0.19; *p* = 0.01; adjusted *R*^2^ = 0.03) but was not associated with frequency of OBEs (*ß* = −0.11; *p* = 0.14) or SBEs (*ß* = −0.12; *p* = 0.12). These findings remained consistent in the full models controlling for covariates (Table 2).

**Table 2:** Full models exploring the relation between positive affect and binge eating episodes.

Predictors	*B*	Std. error	*ß*	*t*	*p*	VIF
*Total binge episodes*						
Positive affect	−0.76	0.35	−0.19	−2.15	0.03*	1.17
Negative affect	−0.29	0.34	−0.07	−0.85	0.40	1.11
Race	12.17	6.01	0.18	2.03	0.045*	1.18
Ethnicity	−7.66	7.57	−0.09	−1.01	0.31	1.14
Gender	−8.94	6.64	−0.11	−1.35	0.18	1.04
Income	0.00	0.00	−0.02	−0.28	0.78	1.04
Unemployment status	−18.46	9.05	−0.18	−2.04	0.04*	1.09
*Objective binge episodes*						
Positive affect	−0.58	0.31	−0.16	−1.84	0.07	1.17
Negative affect	−0.07	0.31	−0.02	−0.21	0.83	1.11
Race	11.47	5.35	0.19	2.15	0.03*	1.18
Ethnicity	−12.48	6.74	−0.16	−1.85	0.07	1.14
Gender	4.59	5.91	0.07	0.78	0.44	1.04
Income	0.00	0.00	−0.13	−1.52	0.13	1.04
Unemployment status	−14.21	8.06	−0.15	−1.76	0.08	1.09
*Subjective binge episodes*						
Positive affect	−0.21	0.21	−0.09	−0.99	0.32	1.17
Negative affect	−0.30	0.21	−0.13	−1.47	0.15	1.11
Race	0.01	3.61	0.00	0.00	1.00	1.18
Ethnicity	4.87	4.55	0.10	1.07	0.29	1.14
Gender	−10.40	3.99	−0.22	−2.61	0.01**	1.04
Income	0.00	0.00	0.06	0.66	0.51	1.04
Unemployment status	−2.70	5.44	−0.04	−0.50	0.62	1.09

*p ≤ 0.05; **p ≤ 0.01.

There was a significant difference in average positive affect scores between people who endorsed total binge eating above versus below the clinical threshold (*t* = 2.76; *p* = 0.01), with lower positive affect scores observed among people who endorsed ≥12 episodes of binge eating regardless of episode size. However, these findings did not hold in evaluations of average positive affect scores between people who endorsed OBEs (*t* = 0.41; *p* = 0.68) or SBEs (*t* = 0.71; *p* = 0.48) above versus below the clinical threshold.

Finally, there was a significant difference in average total binge episodes among people with low versus higher positive affect (*t* = 2.52; *p* = 0.01), with more binge episodes observed among people with low positive affect. However, these findings did not hold in evaluations of average OBEs (*t* = 0.997; *p* = 0.32) or average SBEs (*t* = 1.83; *p* = 0.07) among people with low versus higher positive affect.

## Discussion

Low positive affect has been theorized to directly and indirectly increase binge eating (Mason et al., 2021a). The present study aimed to contribute to the literature on this theory by exploring whether low positive affect is associated with binge eating characteristics, namely binge episode frequency and size.

Consistent with Mason et al.’s (2021a) model, we found that lower positive affect was associated with more frequent binge eating, regardless of episode size, including when controlling for other factors associated with binge eating (e.g., socio-demographic characteristics, negative affect) and when comparing individuals who endorsed the lowest levels of positive affect to those with higher levels of positive affect. However, these findings did not hold when exploring OBEs and SBEs separately or when evaluated in comparison to each other. This may be because the number of OBEs or SBEs was smaller than total binges (by definition), making it more challenging to detect differences with the smaller spread. Our latter results contrast with prior research that have shown associations between positive affect and OBEs (Munsch et al., 2012) and SBEs (Van Malderen et al., 2019). One reason for these differences may be our focus on the frequency of binge episodes, whereas previous studies have examined the presence or absence of binges and over shorter durations (Munsch et al., 2012; Smith et al., 2019; Van Malderen et al., 2019; Schaefer et al., 2020). Indeed, because our evaluation included a broader spectrum of binge eating (e.g., size, frequency duration) than previous studies, our findings are an important contribution to the research in this area because they suggest that low positive affect may be an important target for those experiencing both small and large binge episodes, rather than one or the other; this is consistent with the notion that loss of control, not binge size, is the core feature of binge eating (Fitzsimmons-Craft et al., 2014).

Our study adds to the growing literature on the importance of targeting positive affect in the treatment of binge eating (Mason et al., 2021a). To date, theoretical models and treatments for binge eating disorder, and other psychiatric disorders, have focused on feelings of negative affect (Fairburn, 2008; Rieger et al., 2010; Craske et al., 2016; Dingemans et al., 2017), yet emerging data suggest there may be benefits to improving positive affect. For example, in the treatment of mood and anxiety disorders, upregulating positive affect was more effective than downregulating negative affect (Craske et al., 2019); this notion is being explored in eating disorders like anorexia nervosa (Coniglio et al., 2019; Haynos et al., 2021) and may extend to other eating disorder behaviors like binge eating. Indeed, increasing positive affect may be an important addition to binge eating treatment plans given that positive affect predicts BED treatment outcomes (Mason et al., 2021b,c) and has been theorized in the maintenance of binge eating. However, because our study was conducted using data only collected at one time point, future research should extend this work on binge characteristics that are assessed over clinically-relevant durations in longitudinal analyses to understand how this relation changes with time. Moreover, given our findings on the unique significant effect that race and unemployment status had alongside positive affect on total binge episodes, future studies should also explore the part these factors play in the process of establishing the role of increasing positive affect in clinical treatment plans.

### Strengths & limitations

The strength of this study is our evaluation of a large, diverse treatment-seeking sample of adults who engaged in recurrent binge eating of different episode types based on clinical assessment. However, limitations should be noted. Our study is an exploratory secondary analysis and was not powered to detect effects for this research question. Future studies should evaluate these research questions in a sample with greater statistical power to test these relationships. Additionally, although previous studies also have utilized versions of the PANAS to assess for risk of binge eating amongst varying levels of affect (e.g., Smith et al., 2019; Van Malderen et al., 2019; Schaefer et al., 2020), we suggest that future research consider using validated measures of emotion dysregulation like the difficulties in emotion regulation scale (Weiss et al., 2015), which may better capture positive emotion dysregulation. Relatedly, as a cross-sectional analysis, we were unable to test how changes in affect impact binge eating, as proposed in Mason et al.’s theoretical model (Mason et al., 2021a). Additional limitations include targeting only individuals with obesity. Despite that binge eating disorder is most common among those with higher weight statuses (Udo and Grilo, 2018), future research should seek to include individuals across the weight spectrum who engage in binge eating. The inclusion of a treatment-seeking sample may have created a sampling bias and therefore influenced the overall levels of positive affect, as individuals seeking treatment for binge eating may have lower positive affect levels as compared to those not seeking treatment. Conversely though, this adds strength to our study’s clinical validity regarding the levels of positive affect we might expect to see among individuals with binge eating presenting for treatment. Although we used a variety of recruitment sources (to reflect different potential implementation methods for a digital intervention, such as, as an online service, within healthcare settings, and as a public health initiative), we were unable to evaluate differences in the yield of those individual sources and therefore cannot determine its impact on the results of this study. Lastly, our sample only included individuals who engaged in binge eating. Although this is a point of novelty compared to prior research that primarily has focused on comparisons in positive affect between those who endorse versus do not endorse binge eating, we cannot similarly compare levels of positive affect in our sample.

## Conclusion

Overall, this study explored the association between low positive affect and binge eating characteristics (frequency, size), and results lend support for the theory that low positive affect is associated with binge eating. Although our analyses were exploratory in nature, findings suggest that increasing positive affect may be an important treatment consideration for those with recurrent binge eating and lends further support for future research in this underexplored area of positive affect and binge eating.

## Data availability statement

The original contributions presented in the study are included in the article/supplementary material, further inquiries can be directed to the corresponding author.

## Ethics statement

The studies involving human participants were reviewed and approved by Northwestern University Institutional Review Board. The patients/participants provided their written informed consent to participate in this study.

## Author contributions

RF, TM, and AG contributed to the conception and design of the study and performed the statistical analysis. RF and AG wrote the first draft of the manuscript. JK wrote sections of the manuscript. All authors contributed to the article and approved the submitted version.

## Funding

This research was supported by the National Institute of Diabetes and Digestive and Kidney Diseases (K01 DK116925).

## Conflict of interest

The authors declare that the research was conducted in the absence of any commercial or financial relationships that could be construed as a potential conflict of interest.

## Publisher’s note

All claims expressed in this article are solely those of the authors and do not necessarily represent those of their affiliated organizations, or those of the publisher, the editors and the reviewers. Any product that may be evaluated in this article, or claim that may be made by its manufacturer, is not guaranteed or endorsed by the publisher.
